# Supplementary material for: Prefrontal cortex astroglia modulate anhedonia-like behavior
Source: Mol Psychiatry. 2023 Sep 11;28(11):4632–41. doi: 10.1038/s41380-023-02246-1 (PMC10914619; doi:10.1038/s41380-023-02246-1)
Supplement: Supplementary file 2 — Supplementary Figures with Titles [file 41380_2023_2246_MOESM2_ESM.pdf]

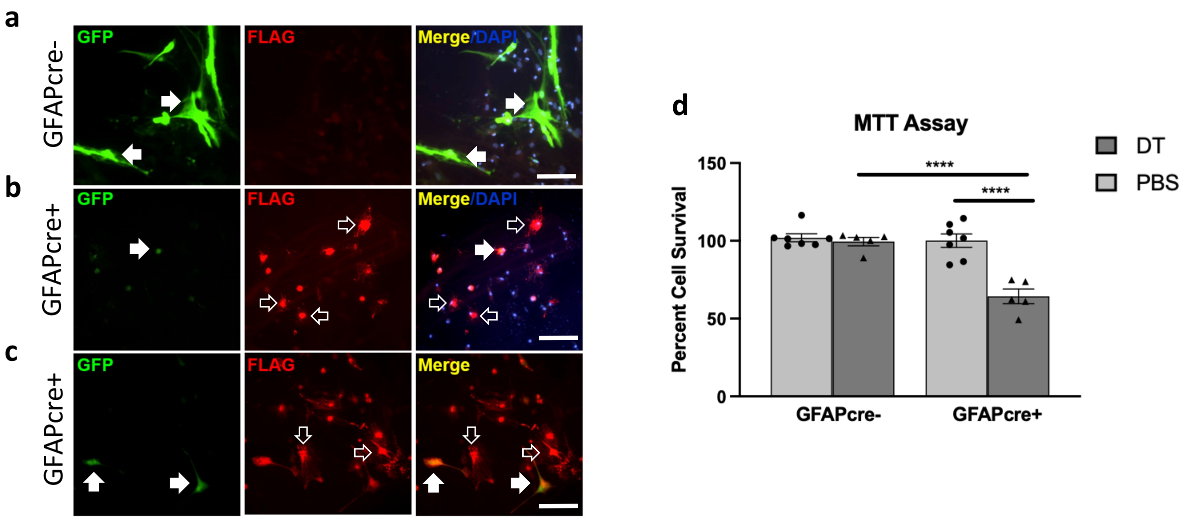

Fig. S1 – Conditional expression of diphtheria toxin receptor (DTR) and cell ablation in primary astrocyte culture from glial fibrillary acidic protein (GFAP) cre+ and cre- mice.

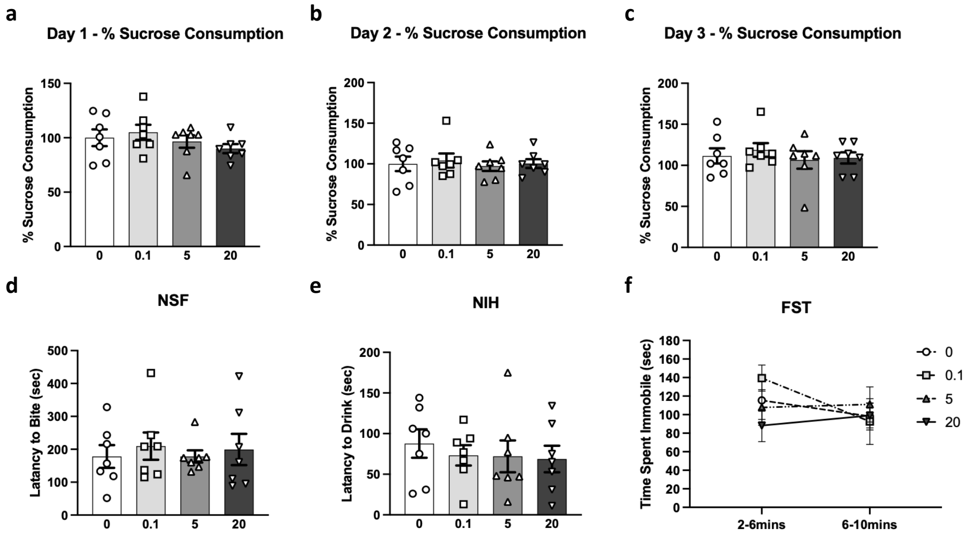

Fig.S2: Effects of cortical infusion of AAV5-GFP-DIOCMV-DTRflag and i.p. injections of diphtheria toxin (DT) in GFAPcre- mice.

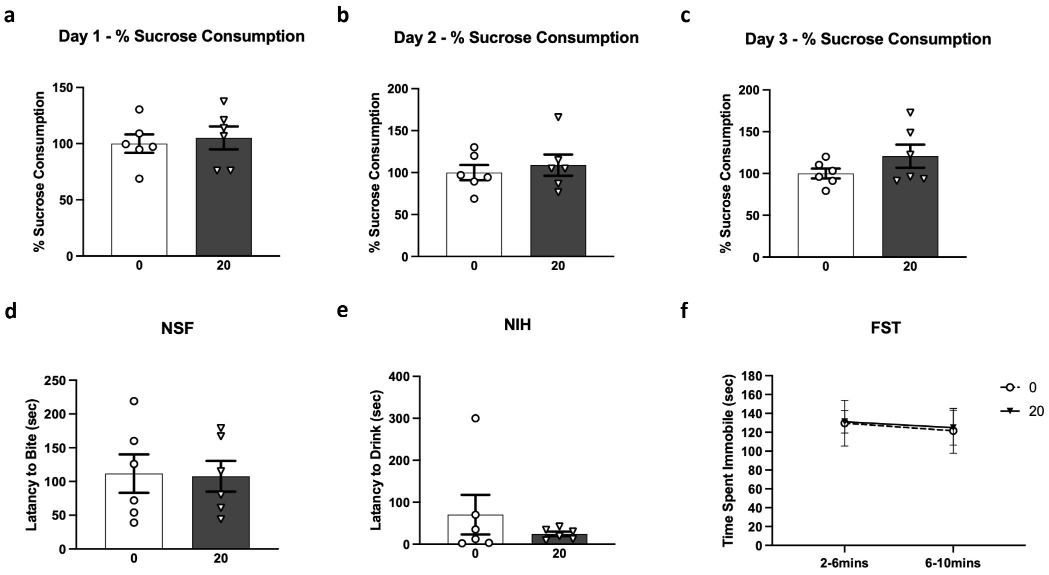

Fig.S3: Effects of cortical infusion of AAV5-GFP-mDIOCMV-DTRflag and i.p. injections of diphtheria toxin (DT) in GFAPcre+ mice.

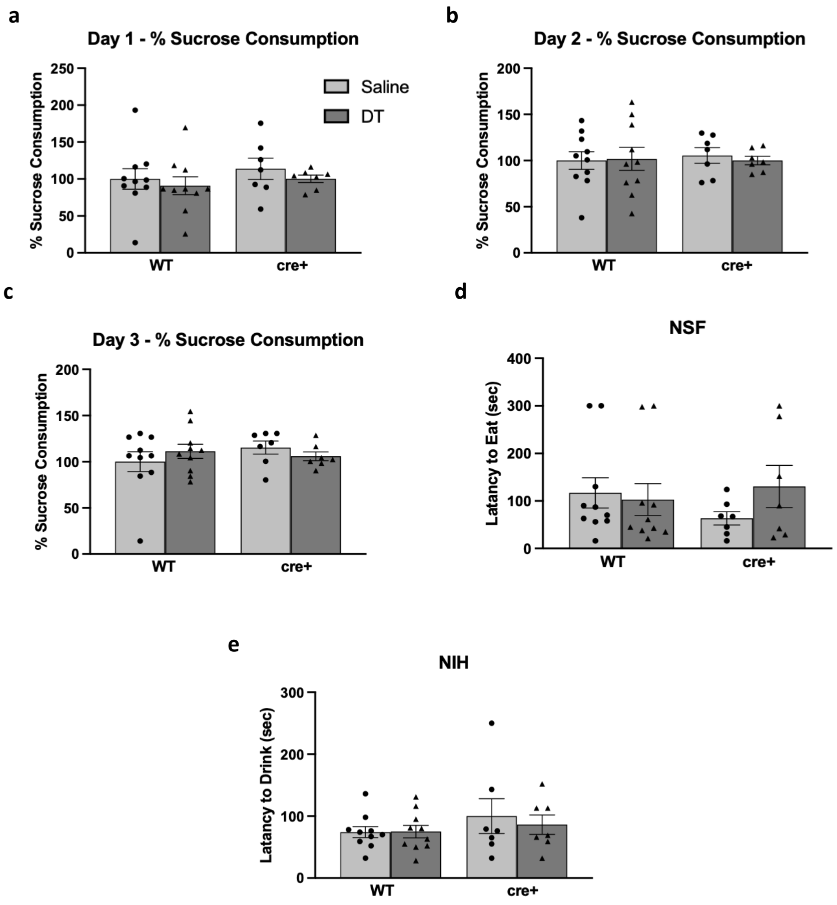

Fig.S4: Effects of striatal infusion of AAV5-GFP-DIOCMV-DTRflag and i.p. injections of diphtheria toxin (DT) in GFAPcre+ and cre- mice.

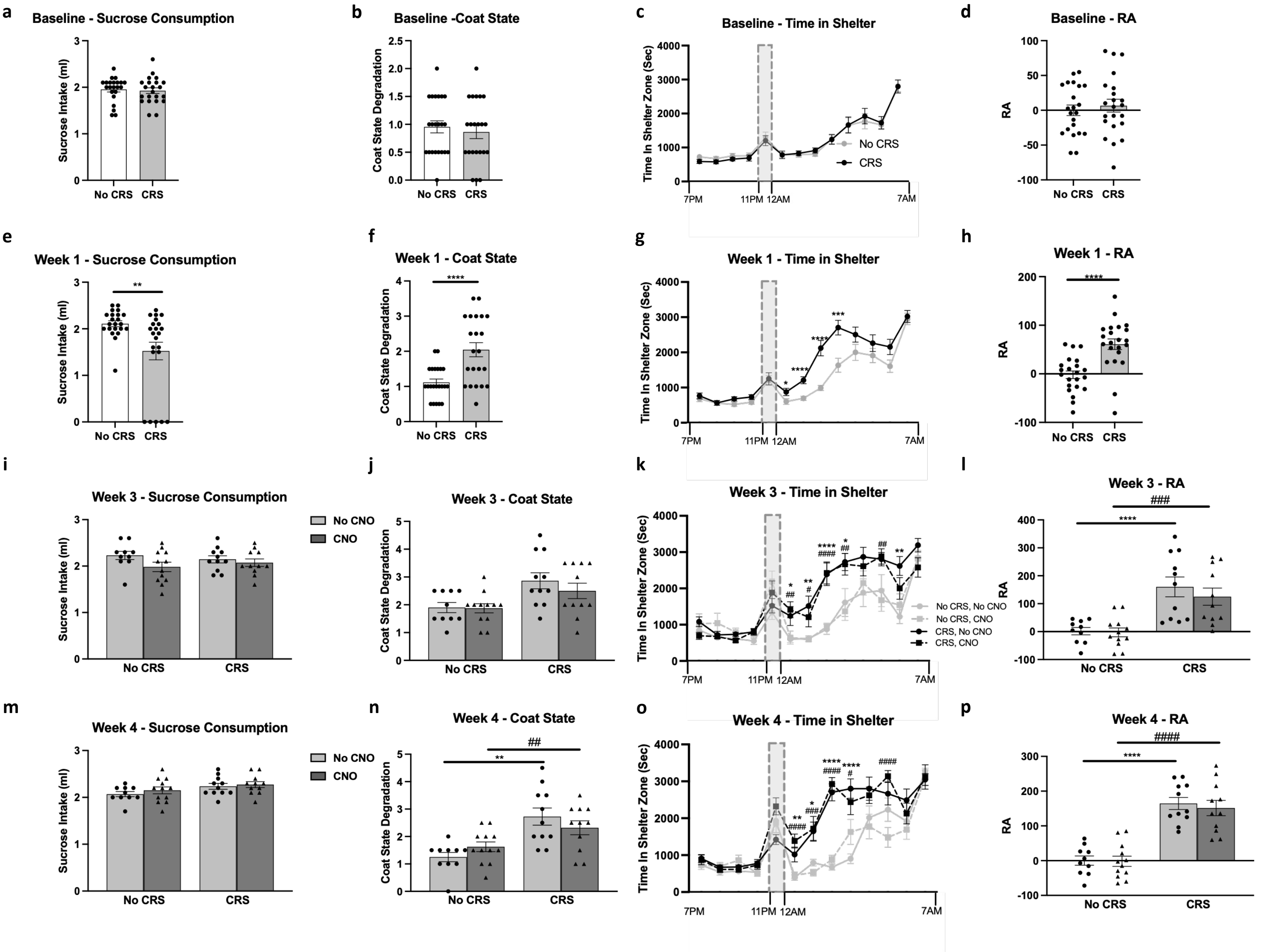

Fig.S5: Cortical Glial Fibrillary Acidic Protein (GFAP)+ cell activity enhancement reverses chronic restraint stress (CRS)-induced anhedonia- but not anxiety-like behavior

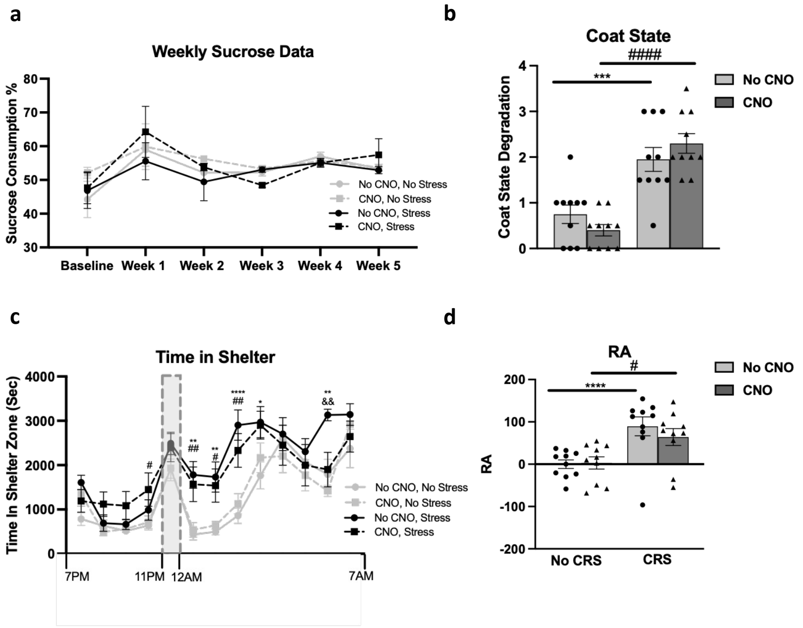

Fig.S6: Chronic restraint stress (CRS) and chronic clozapine-N-oxide (CNO) administration in C57Bl/6.

a

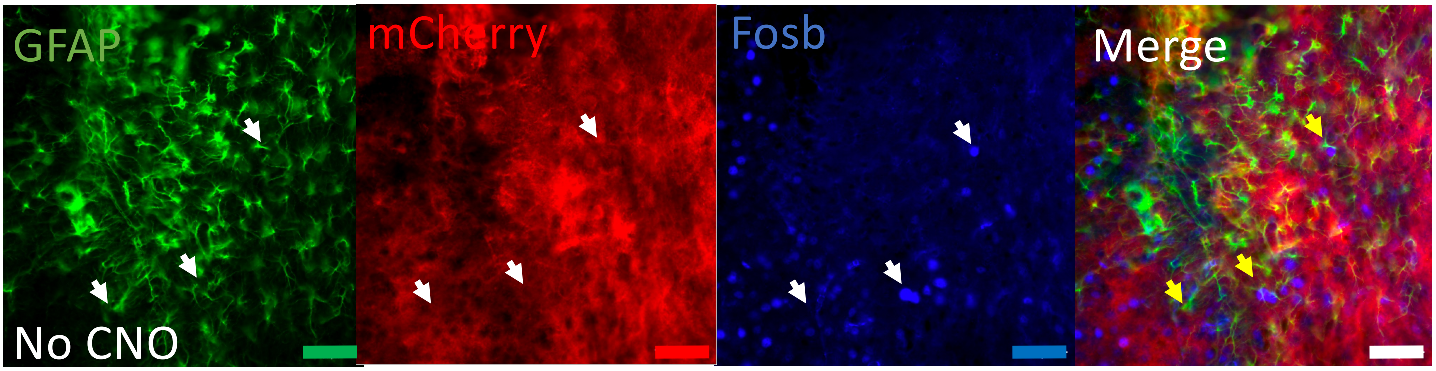

b

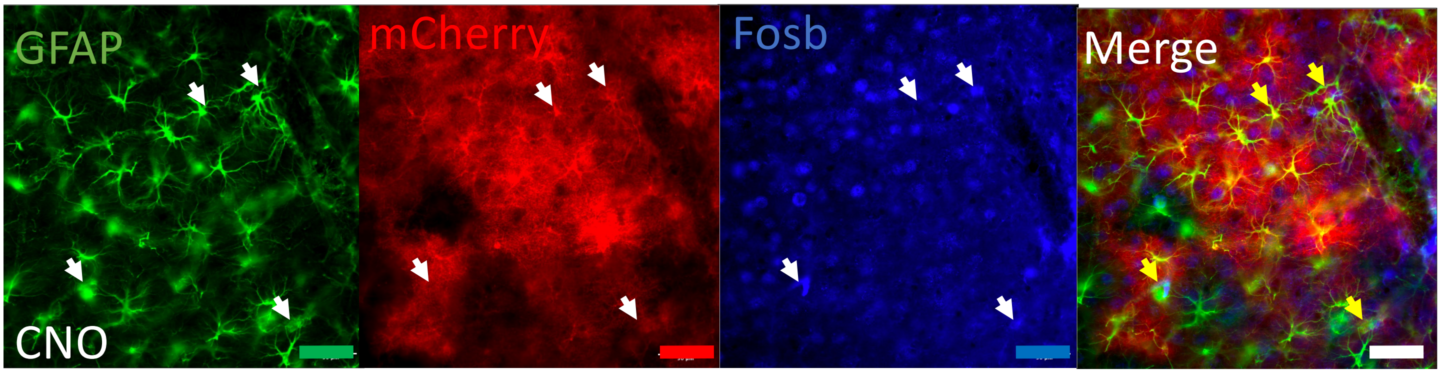

Fig.S7: Increased Fosb intensity in infected Cortical Glial Fibrillary Acidic Protein (GFAP)+ cells following CNO administration.
